# Creating a mental health talanoa to promote a collaborative approach to wellbeing across Pacific peoples

**DOI:** 10.1177/10398562241281576

**Published:** 2024-09-23

**Authors:** Jioji Ravulo

**Affiliations:** Sydney School of Education and Social Work, 4334 The University of Sydney, Sydney, NSW, Australia

**Keywords:** talanoa, Pacific communities, health literacies, Indigenous wellbeing, service provision

## Abstract

**Objective:**

Promoting holistic health and wellbeing is a shared conversation, or talanoa, requiring collaboration between individuals, their families and wider communities. This paper will explore various community-based initiatives privileging Pacific epistemologies and ontologies that promote and provide accessible resources improving mental health literacies.

**Method:**

Three specific initiatives are discussed in this paper; Mental Health Talanoa (MHT), Open Worksheet and Wellbeing Talanoa. These provide a platform to understand practical ways to support Pacific peoples in various contexts.

**Results:**

The MHT project offers a nuanced understanding of symptomatology related to common mental disorders amongst Pacific peoples, a nuanced understanding of the barriers and enablers to health literacies and help seeking behaviour, and a series of infographics, including the Pacific Mental Health Lexicons (PIMHL). The Open Worksheet is a dynamic tool underpinned by a dialogical and relationally driven way to understanding individual and familial narratives. The Wellbeing Talanoa supports a communally orientated opportunity to enhance a sense of connection to self and others whilst therapeutically reviewing social and welfare needs and solutions.

**Conclusion:**

Developing and implemented Pacific approaches that are grounded in Pacific values and practices can lead to enhanced help seeking behaviour, engagement, service retention and provision.

## Pacific wellbeing through talanoa

Pacific peoples and their wellbeing are inextricably connected to self and others. Their ability to thrive socially and emotionally is based on a reciprocal approach to life, where ‘the need to cater for other’s wellbeing is embedded in our shared consciousness that we live not just to support our own wellbeing, but the wellbeing of others in and around the space in which we occupy’.^1^ This underlying notion of space in various indigenous Pacific languages is known as *Vā*, a sacred space, that is nurtured between individuals, families and the wider community. Failure to nurture the Vā creates dissonance and a lack of connection to self and others. Therefore, promoting initiatives that support and privilege Pacific epistemologies and ontologies on wellbeing is vital. This can assist in enabling a holistic view of wellbeing, ‘which ensure people are in right relationships with each other in and across families and communities’.^1^

To nurture the Vā, a *talanoa* approach is commonly utilised. A talanoa, by definition, is to hold space to enact a collaborative and shared conversation, where individuals are provided with the opportunity to create a safe space with each other to dialogically explore and talk about anything, everything, or nothing at all. Like *yarning circles* undertaking by First Nations Australians, or *korero* by Māori people in Aotearoa New Zealand, a talanoa provides opportunities to connect and form community, nurturing the Vā amongst self and others.

This paper will explore three specific collaborative initiatives that were created to nurture Vā using a talanoa approach amongst Pacific communities across Oceania. Each is underpinned by the importance of understanding the individual in their wider social context, whilst meaningfully integrating Pacific perspectives and practices into service models that in turn strive to improve health literacies, leading to enhanced help seeking behaviour and service engagement.

## Three Pacific initiatives

Each of the three initiatives listed below provides synergies between the concept of Vā, nurturing space, and talanoa, creating a shared conversation. Scholarly research and writing have been undertaken on each initiative and is utilised to support a shared understanding of meaningfully including Pacific perspective and practices across mental health spaces and places Table 1.

**Table 1.** Summary of three initiatives.

Name	Purpose	Outputs	Outcomes
*Mental Health Talanoa*	Promote health literacies and help seeking behaviours amongst Pacific communities	• Quantitative survey data • Qualitative talanoa data	• Infographics • YouTube videos • Critical conversations
*The Open Worksheet*	Developing inter and intra personal reflective skills and practices	• Open worksheet tool • Dialogue/talanoa	• Insights into thoughts, feelings and behaviours • Goals for ongoing development
*Wellbeing Talanoa*	Creating safe spaces to undertake a talanoa on wellbeing	• Collective conversation with peers • Reflective space	• A community of care amongst group • Self-care options

The *Mental Health Talanoa (MHT)*^2^ initiative strives to understand the prevalence, morbidity and health seeking behaviours related to mental health within the Pacific diaspora in Australia and beyond. The research project was funded by the New South Wales (NSW) Ministry of Health alongside three Primary Health Networks (PHN’s) – Western Sydney PHN, South Western Sydney PHN, and Nepean Blue Mountains PHN – from 2019 to 2021. The MHT model created a four phased approach known as the FRET model: **F**ormation (Community Engagement), **R**esearch (Community Collaboration), **E**ducation (Community Resources) and **T**raining (Community Development). Each phase of the model promoted direct interactions with the Pacific community, nurturing the Vā, and ensuring Pacific perspective and practices were included throughout. Each phase also acted as a ripple to inform each other, like a fret – a small wave. Within the research, a mixed method approach was undertaken. The WHO Self Reporting Questionaire (SRQ-20) profiled possible symptomatology specific to Pacific people experiencing a common mental disorder (CMD). Qualitatively, a series of talanoa sessions were undertaken with Pacific people residing in greater western Sydney. Key themes from both methods were then utilised to create a series of tools and resources to further support a mental health talanoa – a shared conversation amongst Pacific people and their health practitioners in developing health literacies alongside help seeking behaviour and service engagement. The importance of building capacity within the health workforce was also emphasised, ensuring that a shared responsibility was enacted across the community, both Pacific and non-Pacific. A unique offering and resource came through the development of the Pacific Indigenous Mental Health Lexicons (PIMHL), a series of infographics that provided translations of common mental health phrases and terms in four Pacific indigenous languages; Bislama (Vanuatu), Fijian (Fiji), Samoan (Samoa) and Tongan (Tonga). Translations were supported by Pacific mental health practitioners from each of the Pacific Islands, enabling a regional approach to the research, and its practical applications.

The *Open Worksheet*^3-5^ is a dialogically driven therapeutic tool designed to provide a reflection on key topic areas. Developed by Ravulo J^6^ during work with Pacific young people involved in the criminal justice system, this simple approach enables people of all ages to develop insights into their thoughts, feelings and behaviours. An opportunity to also discuss their perceived narratives, alongside their motivations underpins the tool. Three counselling modalities – cognitive behavioural therapy, narrative therapy, and motivational interviewing – are interwoven in this conversational process. Participants will take a blank sheet of paper, and on one side draw an oval with a single line underneath. On the other side, they will draw a line in the middle of the page. Turning back to the front to start the activity, they will be asked to reflect on a particular question, and then respond by drawing their facial expression in the oval, along with naming the feeling by using an adjective they nominate in response to the question. They list the feeling word on the single line below the oval. Questions can include ‘how do you feel about your future?’, ‘how do you feel about your family?’, ‘how do you feel about school?’. Each question is designed to help participants reflect on their current journey based on these topic areas. After they have completed this initial part of the activity, they then turn to the other side of the sheet and start to dot point ‘why’ they responded that way to the reflective question. This then provides participants with an opportunity to unpack, and further develop a narrative around their situation and circumstances. After this is then listed, and discussed in detail, the final part of the activity is to dot point ‘what’ needs to be done to achieve possible change or maintain their current position. This then enables an exploration of motivation to enact this process moving ahead. By utilising the *Open Worksheet* to create a talanoa as a therapeutic process, participants can dialogically and relationally explore the Vā in which they are located, and the possibilities based on their responses.

A *Wellbeing Talanoa*^7^ is a group work activity that supports and creates a sense of connection and community to self and others. It enables individuals to see themselves as part of something bigger, and to explore solutions to presenting social and welfare needs. The group is set up as circle, with all participants welcomed with an overview of the 1.5-h session being facilitated as a talanoa – a safe space to create a shared and supportive conversation about anything, everything, or nothing at all. Where there is no right or wrong answer, and everyone will be encouraged to participate around the circle. There are four phases to the talanoa. Firstly, each person must *check in* by introducing themselves through the following four questions – ‘i) Name, (ii) Heritage, (iii) Affiliation,/Vocation, and (iv) How are you doing?’ This initial phase is to provide an opportunity for people to know who’s in the space, and to orient the talanoa as a safe space where everyone is engaged from the beginning. Secondly, the *further exploration* phase continues the circle conversation by asking each participant to reflect and share their own response to the following broader question ‘What’s happening?’ (based on their initial response to ‘how are you doing?’). As responses are shared around the circle, it provides an opportunity for the facilitator to pause on a particular person’s contribution and ask the broader group ‘Do other people relate?’ and ‘What could assist’. This is where participants are provided an opportunity to nurture the Vā as a sense of connection to each other, exploring possible solutions to such challenges. Where people are encouraged to locate their individual struggles in a wider context with view to promote a level of hope to possibly overcome such concerns. After everyone has had a turn to respond, phase three is *wellbeing discussion* where in pairs, participants are given 5 minutes to discuss ‘what will you do to improve your wellbeing?’. This provides an opportunity to think of practical ways to enact self-care. Finally, the fourth phase, *check out* brings the talanoa to an end, where each person, once again in order around the circle, has to individually share their responses to three last quick reflective questions; (i) what is your wellbeing activity? (ii) how are you feeling off the back of the talanoa? and (iii) a takeaway from the group? This promotes a broader sharing and profiling of the practical self-care activities, an opportunity to ensure people are emotionally okay after participating in this group, and an insight into the possible benefits of being involved in the talanoa. After each person shares, a *cobo* (a cupped hand clap – pronounced *thombo*) is given to express a sign of appreciation for their participation, furthering a sense of community and connection to self and others.

## Conclusion

Culturally safe and sustainable approaches in supporting mental health and wellbeing when working holistically with Pacific people’s needs to be nuanced with Pacific practices and perspectives. By utilising a talanoa, a safe space to engage in a dialogical and relationally driven way, it provides a platform for the Vā, the sacred space between us, to be nurtured. As a result, Pacific people are afforded tangible scope to improve their health literacies, leading to enhanced help seeking behaviour, engagement, service retention and provision. In turn, it enables an individual to collaboratively explore and effectively respond to where *they are at* in their own journey, as part of a broader connection to their families and wider community.
